# Anti-Inflammatory Effects of M-MSCs in DNCB-Induced Atopic Dermatitis Mice

**DOI:** 10.3390/biomedicines8100439

**Published:** 2020-10-21

**Authors:** Bokyeong Ryu, Jieun Baek, Hana Kim, Ji-Heon Lee, Jin Kim, Young-Hoon Jeong, Seul-Gi Lee, Kyu-Ree Kang, Min-Seok Oh, Eun-Young Kim, C-Yoon Kim, Hyung Min Chung

**Affiliations:** 1 Department of Laboratory Animal Medicine, College of Veterinary Medicine, Seoul National University, Seoul 08826, Korea; hobitmilk@snu.ac.kr (B.R.); jk208019@snu.ac.kr (J.K.); 2 Department of Stem Cell Biology, School of Medicine, Konkuk University, Seoul 05029, Korea; jieuns2322@konkuk.ac.kr (J.B.); flower216@konkuk.ac.kr (H.K.); lzh911@konkuk.ac.kr (J.-H.L.); panthera@konkuk.ac.kr (Y.-H.J.); maxwisdom@konkuk.ac.kr (S.-G.L.); krkang@konkuk.ac.kr (K.-R.K.); oms860531@konkuk.ac.kr (M.-S.O.); 3 Advanced Analysis Center, Korea Institute of Science and Technology (KIST), Seoul 02792, Korea; 4 Mireacellbio Co., Ltd., Seoul 04795, Korea; jlokey@miraecellbio.com

**Keywords:** embryonic stem cell (ESC), mesenchymal stem cell (MSC), atopic dermatitis (AD), inflammation, treatment

## Abstract

Atopic dermatitis (AD) is an inflammatory skin disease caused by an imbalance between Th1 and Th2 cells. AD patients suffer from pruritus, excessive dryness, red or inflamed skin, and complications such as sleep disturbances and depression. Although there are currently many AD treatments available there are insufficient data on their long-term stability and comparative effects. Moreover, they have limitations due to various side effects. Multipotent mesenchymal stem cells (M-MSCs) might have potential for next-generation AD therapies. MSCs are capable of immune function regulation and local inflammatory response inhibition. M-MSCs, derived from human embryonic stem cells (hESC), additionally have a stable supply. In L507 antibody array, M-MSCs generally showed similar tendencies to bone marrow-derived mesenchymal stem cells (BM-MSCs), although the immunoregulatory function of M-MSCs seemed to be superior to BM-MSCs. Based on the characteristics of M-MSCs on immunoregulatory functions, we tested a M-MSC conditioned media concentrate (MCMC) in mice with AD lesions on their dorsal skin. MCMC significantly decreased RNA expression levels of inflammatory cytokines in the mouse dorsal skin. It also suppressed serum IgE levels. In addition, significant histopathologic alleviation was identified. In conclusion, secretions of M-MSCs have the potential to effectively improve AD-related inflammatory lesions. M-MSCs showed potential for use in next-generation AD treatment.

## 1. Introduction

Atopic dermatitis (AD) is a chronic inflammatory skin disease caused by an imbalance in immune responses. It is a disease that requires the development of effective treatments [1]. AD usually begins in infancy and extends into adult life with chronic or recurrent persistency. The prevalence of adult persistent AD increases with an increased incidence of childhood AD [2,3]. AD patients complain of discomforts such as pruritus (54.4%), active dryness or scaling (19.6%), and red or inflamed skin (7.2%) [4]. Such discomfort causes inconvenience and a number of complications that can decrease the quality of life such as sleep disturbances, anxiety, depression, obesity, poor cardiovascular function, and extracutaneous infections. 

Inflammatory lesions of AD are induced by allergic reactions caused by an imbalance between Th1 and Th2 cells [5]. Th1 immune response is involved in infection while the Th2 immune response is involved in response to stimulation [6]. When Th2 cells are activated, cytokines such as IL-4, IL-5, IL-10, and IL-13 are secreted to enhance the humoral immune response [7]. IL-4 can also inhibit the Th1 cell function, causing a Th2 dominance and Th1/Th2 imbalance. In addition, the mast cell-dependent histamine response can be enhanced by IL-4 [8,9]. Mast cells are major effector cells of immunoglobulin E (IgE)-mediated hypersensitivity reactions. Histamine secretion can also promote the production of IgE. Eventually, the overproduction of serum IgE could be caused by IL-4 in AD [10]. Therefore, IL-4 should be controlled to improve AD symptoms. However, conventional therapies for AD such as mid- to high-potent corticosteroids and/or calcineurin inhibitors [11] or antihistamines [12] have been reported to have side effects including systemic immune suppression, metabolic abnormalities, viral infections, skin atrophy, and cognitive decline [13]. Therefore, there is an urgent need to develop new therapies for AD with not only efficacy but also broadened safety margins. 

In recent years, stem cell therapies have opened new avenues for the treatment of some refractory diseases. Mesenchymal stem cells (MSCs) are adult stem cells isolated from a wide range of tissues, including bone marrow, adipose tissue, dental pulp, and umbilical cord blood [14]. MSCs can interact with innate and adaptive immune systems and regulate the proliferation, differentiation, and activation of immune cells including T cells, B cells, dendritic cells, and natural killer cells [15]. They can also regulate B lymphocyte maturation and reduce the degranulation of mast cells [16,17,18], implying their potential as a cell-based therapy for AD. However, their limited in vitro proliferative capacity is one of the major problems limiting their broader clinical applications [19]. Therefore, there is a need to secure stable supply resources for MSCs. 

Human embryonic stem cells (hESC) can be used as a stable resource of MSCs [20]. ESCs established from the inner cell mass of the blastocyst can be differentiated into all possible types of cells that can be expanded in vitro immortally, resulting in unlimited self-renewal. Due to these characteristics, hESC-derived multipotent MSCs (M-MSCs) are one of the attractive candidates for stem-cell-based therapeutics [21,22,23,24]. We thus validated the efficacy of M-MSCs as a cell-based therapy. We firstly analyzed protein expression patterns of M-MSCs in comparison with bone marrow-derived mesenchymal stem cells (BM-MSCs). Following the finding that secretions of M-MSCs were superior as an immunoregulator to BM-MSCs, secretions of M-MSCs were used to treat a mouse model of AD to evaluate gene expression levels and serum IgE levels that might be associated with AD etiology. Histological and immunohistochemical analyses were also performed. The results of this study provide information on whether M-MSCs might possess an anti-inflammatory effect in a mouse model of AD and their possibility for use as next-generation cell-based therapeutics.

## 2. Materials and Methods

### 2.1. Chemicals and Reagents

DMEM/F-12 Medium, knockout serum replacement, glutamine, β-mercaptoethanol, nonessential amino acids, and human recombinant bFGF were purchased from Invitrogen Corporation (Carlsbad, CA, USA). EBM^TM^-2 Basal Medium and EGM^TM^-2 MV Microvascular Endothelial Cell Growth (EGM2-MV) Medium SingleQuots^TM^ supplements were purchased from Lonza (Basel, Switzerland). Trypsin-EDTA (0.25%) was purchased from Gibco (Waltham, MA, USA). Sodium chloride, 1-chloro-2,4-dinitrobenzene (DNCB), eosin Y, toluidine blue O, hematoxylin, and hydrochloric acid were purchased from Sigma-Aldrich (St. Louis, MO, USA). Total RNA Extraction Kit was purchased from iNtRON (Gyeonggi, Korea). High Capacity cDNA Reverse Transcription Kit was purchased from Thermo Fisher Scientific (Waltham, MA, USA). FastStart Essential DNA Green Master and serum-containing medium were purchased from Roche (Basel, Switzerland). Ammonia was purchased from JUNSEI (Chuo-ku, Tokyo). Anti-F4/80 antibody was purchased from Santa Cruz Biotechnology (Dallas, TX, USA). Mouse IgE ELISA Set was purchased from BD (Franklin Lakes, NJ, USA).

### 2.2. hESC Culture

Undifferentiated hESC line SNUhES was cultured as previously described [24]. The hESCs between passage 40–60 were used. The hESCs were cultured in DMEM/F-12 medium supplemented with 20% knockout serum replacement, 1 mM glutamine, 0.1 mM β-mercaptoethanol, 0.1 mM nonessential amino acids, and 4 ng/mL human recombinant bFGF at 37 °C in 5% CO_2_ and 95% humidity.

### 2.3. Isolation of M-MSCs and Culture

For embryoid body (EB) formation, hESC colonies were removed from the feeder layers by dispase treatment (1 mg/mL in a serum-containing medium; Roche, Basel, Switzerland). The harvested hESC colonies were grown in suspension culture for 2 days with the same hESC culture medium except for bFGF. The porous membrane transwell inserts with 8 μm pores were used to isolate MSC-like cells. The upper compartment of the inserts was coated with 0.1% gelatin, and EBs were attached in EGM2-MV medium for 5 days. The migrated cells to the lower compartment of the inserts formed colonies, which were gently scraped and subcultured onto a new 100 mm dish in the same media. M-MSCs were cultured in EGM2-MV medium at 37 °C in a humidified atmosphere with 5% CO_2_. Cells were expanded for fewer than 10 passages to ensure that multipotency was preserved. The medium was changed every two days.

### 2.4. M-MSC Cultured and Conditioned Media Concentration

M-MSCs were seeded in a 100 mm dish at 2.75 × 10^5^, and conditioned media were obtained when the cells on the fourth day were approximately 80% confluent. Ten milliliters of the media was put into a 100 mm dish every time and changed every 2 days. The M-MSC conditioned media and the naïve media were centrifuged for 30 min at 5000 *g* with Macrosep Advance Centrifugal Devices and Omega Membrane 100K (Pall, NY, USA) to obtain M-MSC conditioned media concentrate (MCMC) and naïve media concentrate.

### 2.5. Antibody-Based Protein Microarray

Supernatants and lysates of M-MSCs were compared with those of BM-MSCs and human dermal fibroblasts (hDFs) using a semi-quantitative protein antibody array chip L507 (RayBiotech Inc., Norcross, GA, USA). Each cell was seeded in a 100 mm dish at 3 × 10^5^, and conditioned media and cells were obtained when the cells on the fourth day were approximately 90% confluent. This L507 antibody array was chosen to detect 507 proteins based on biotin labeling. For detection, proteins (50 to 200 μg) were extracted and protein qualities were checked with a BCA protein assay kit (Abcam, Cambridge, UK). Proteins were biotin-labeled followed by protein hybridization according to the manufacturer’s instructions. Fluorescent images were visualized with a streptavidin-cyanine3 conjugate and scanned with a GenePix 4100A Microarray Scanner (Molecular Devices Inc., San Jose, CA, USA). Data were analyzed with a GenePix Pro 7.0 software (Molecular Devices Inc., San Jose, CA, USA). All data were quantile normalized.

Hierarchical clustering to visualize expression patterns of the results was performed using Spearman’s rank correlation after log2 transformation in TIGR MultiExperiment Viewer (MeV) 4.9.0 (The Institute for Genomic Research, and ArrayAssist software, Stratagene, http://www.tm4.org/mev) [25]. Pearson product-moment correlation coefficient rho (r) and their significance levels were calculated with R 3.6.3 (The R Foundation for Statistical Computing c/o Institute for Statistics and Mathematics, Vienna, Austria) using cor() [26,27,28] and rcorr() functions, and were visualized with chart.Correlation() function. To determine the strength of associations, was interpreted as follows: 0.90 to 1.00, very highly correlated; 0.70 to 0.90, highly correlated; 0.50 to 0.70, moderately correlated; 0.30 to 0.50, lowly correlated; and 0.00 to 0.30, negligibly correlated [29]. 

Proteins of M-MSCs/BM-MSCs with a fold change > 1.4 and a *p* value < 0.05 were considered to be differentially expressed proteins (DEPs). Gene Ontology (GO) network and Enrichment Pathway analysis for DEPs were performed using ClueGO plug-in v2.5.4 on Cytoscape v3.7.0 and Java script v1.8.0_211 [30]. ClueGo analysis was incorporated with GO for Biological Process (EBI, UniProt-GOA). Pathway restriction was set to have a *p* value < 0.05. GO tree interval of 4–6 was used to specify GO terms. The connectivity Score (Kappa Score) was set to be 0.4. 

### 2.6. Animals

A total of 35 female 4-week-old BALB/c mice were purchased from ORIENT Bio Inc. (Gyeonggi, Korea). These mice were acclimated for a week and used at the age of 5 weeks old. During the experiment, all mice were provided with tap water and pellets (Altromin, Lage, Germany) ad libitum. All animals were maintained at a temperature of 22 ± 2 °C with a humidity of 55 ± 5% and a light/dark cycle of 12 h/12 h. 

### 2.7. Induction and Treatment of AD-Like Skin Lesions in Mice

AD-like skin lesions were induced according to a previously described method [31] with some modifications. Briefly, 35 mice were shaved of dorsal hair and randomly divided into three groups: (1) MCMC-treated group (called MCMC group, n = 10), (2) naïve media-treated group (called Naïve group, n = 10), (3) only DNCB-treated group (called DNCB group, n = 10), and (4) normal group (n = 5). To induce AD-like skin lesions for 30 mice in the MCMC group, for the Naïve group and the DNCB group 80 μL of 1% (*w*/*v*) DNCB in sunflower oil was applied to the shaved area every other day for two weeks (day 14). Subsequently, they were treated with 80 μL of 0.5% (*w*/*v*) DNCB in the same area every other day for another week (day 21). Meanwhile, mice in the normal group were treated with only sunflower oil for 3 weeks (day 21). After the induction of AD-like skin lesions, the MCMC group was treated with 80 μL/day of MCMC for 10 consecutive days, whereas the Naïve group was treated with 80 μL/day of naïve medium concentrate as a negative control (day 31). At 24 h after the final MCMC or naïve media concentrate treatment, all mice were examined grossly and blood samples were collected under isoflurane anesthesia. Animals were then sacrificed in a CO_2_ chamber and their dorsal skin samples were excised. The DNCB group obtained blood and dorsal skin samples in the same way on day 21, and the normal group was obtained on days 21 and 31.

### 2.8. Histopathological Analysis

To evaluate histopathological changes, the dorsal skin of each mouse was freshly excised and fixed with 10% neutral buffered formalin for 24 h. Tissues were processed using routine tissue techniques and embedded in paraffin. Paraffin-embedded specimens were sliced into 5-μm-thick sections. Sections were then transferred to adhesive microscope slides (Marienfeld, Lauda-Königshofen, Germany). 

Deparaffinized skin sections were stained with hematoxylin and eosin (H&E) or toluidine blue for skin thickness measurement and mast cell detection. Additionally, immunohistochemical staining was conducted using an anti-F4/80 antibody (Santa Cruz Biotechnology, Santa Cruz, CA, USA). All stained sections were then examined with a light microscope Eclipse Ti (Nikon, Tokyo, Japan) to assess histological changes including skin thickening, mast cell infiltration, and F4/80-positive macrophage infiltration. Three sections per animal were used for histological examinations.

### 2.9. Quantitative Real-Time PCR (qPCR)

Total RNA was extracted from the dorsal skin using a Total RNA Extraction Kit (iNtRON, Gyeonggi, Korea). The first-strand cDNA was then synthesized using the extracted total RNA as a template with a High Capacity cDNA Reverse Transcription Kit (Thermo Fisher Scientific, Waltham, MA, USA) according to the manufacturers’ protocols. The resulting cDNA was subjected to qPCR using a FastStart Essential DNA Green Master (Roche, Basel, Switzerland). Primer sequences and detailed experimental conditions for the amplification of GAPDH, IL-1b, IL-4, IL-6, IL-10, and IL-13 are shown in Table 1. All primers were purchased from Macrogen (Seoul, Korea). Cycle threshold (Ct) values from each sample were normalized to those of GAPDH as an internal control (ΔCt = Ct_target gene_ − Ct_GAPDH_). Relative fold changes of target gene expressions were determined using the comparative 2^−ΔΔCt^ method (ΔΔCt = ΔCt_M-MSC_ − ΔCt_control_) [32]. 

### 2.10. Serum Enzyme-Linked Immunosorbent Assay (ELISA)

To determine serum IgE levels, whole blood samples were collected into serum separator tubes. Sera were separated via centrifugation at 12,000 RPM for 10 min and stored at −80 °C until analyzed. Serum IgE levels were measured with a Mouse IgE ELISA Set (BD, Franklin Lakes, NJ, USA). Optical densities were measured at a wavelength of 450 nm.

### 2.11. Statistical Analysis

Data of qPCR are shown as mean ± SEM. Other data are shown as mean ± SD. Differences between groups were assessed by a paired, two-tailed Student’s *t*-test. All statistical analyses were performed using GraphPad Prism v.5 (San Diego, CA, USA). Statistical significance was considered when a *p* value was less than 0.05.

### 2.12. Ethics Statement

All animal experiments were performed in accordance with relevant guidelines and regulations of the Institutional Animal Care and Use Committee of Konkuk University (IACUC authorization no. KU19042, approved at 12 June 2019) accredited for laboratory animal care by the Ministry of Food and Drug Safety of South Korea.

## 3. Results

### 3.1. Protein Antibody Array of M-MSCs

**Figure 1:**
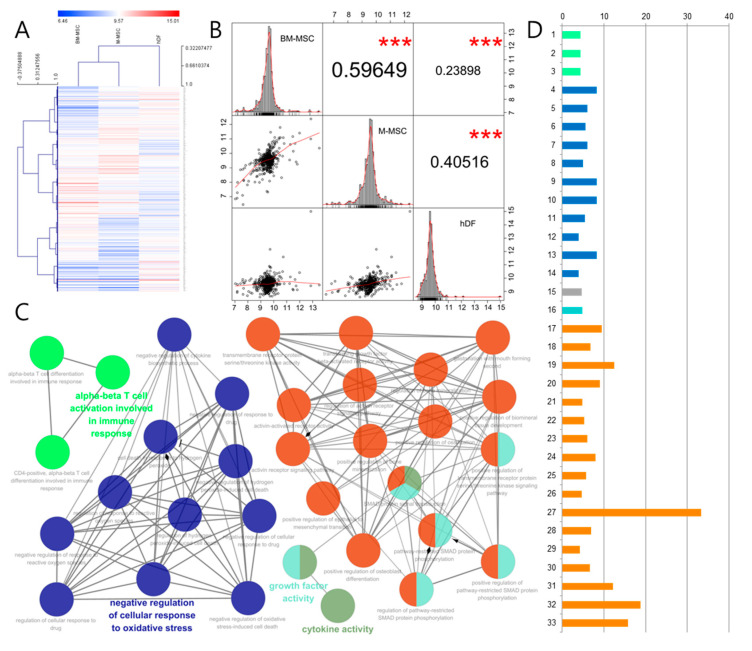
Characterization of M-MSCs (multipotent mesenchymal stem cells) via comparison with bone marrow-derived mesenchymal stem cells (BM-MSCs). M-MSCs and BM-MSCs’ supernatants and lysates were analyzed using L507 antibody array. (**A**) Heatmap of the supernatant of M-MSC, BM-MSC, and human dermal fibroblast (hDF). The color scale at the top represents the expression level, where red, blue, and white colors indicate upregulation, downregulation, and unaltered expression, respectively. (**B**) Correlogram with the significance values between M-MSC, BM-MSC, and hDF. The protein expressions of the cells are scattered as dots and their distribution is shown on the diagonal. Bivariate scatter plots with a fitted line are displayed on the bottom of the diagonal, and the values of the correlation and their significance level are displayed on the top of the diagonal (*p* = 0, ***). Biological processes enriched amongst proteins in M-MSCs than BM-MSCs are shown in a ClueGO enrichment map. (**C**) Each node represents a significantly enriched biological process (*p* < 0.05). (**D**) % genes/term for each node in (**C**) are shown. Features included each node are listed in the Supplementary Materials.

To compare the protein expression profile of M-MSCs with that of BM-MSCs or hDFs, a heatmap was drawn for the whole result from the L507 antibody array (Figure 1A). The dendrite between M-MSC supernatant and BM-MSC supernatant was shorter than one with hDF lysate. With the heatmap results, Pearson product-moment correlation coefficient *r* was calculated. As shown in Figure 1B, The *r* between M-MSCs and BM-MSCs was 0.5964864 with a *p* value of 0. The *r* between M-MSCs and hDFs was 0.4051648 (*p*-value, 0) and the *r* between BM-MSCs and hDFs was 0.2389847 (*p*-value, 5.1210^-8^). Based on the interpretation for *r* (0.70 to 0.90, highly correlated; 0.50 to 0.70, moderately correlated; 0.30 to 0.50, lowly correlated; and 0.00 to 0.03, negligibly correlated) [29], M-MSCs showed a highly moderate correlation with BM-MSCs, while these two kinds of MSCs showed a low correlation or a negligible correlation with hDFs. 

Subsequently, ClueGO analysis was conducted to determine the biological functions of DEPs in M-MSC supernatant compared with BM-MSC supernatant. DEPs in M-MSC lysate were also compared with those in BM-MSC lysate. As shown in Figure 1C, results mainly consisted of two groups: (1) regulation of SMAD protein, and (2) negative regulation of cellular response to oxidative stress. The percentage of associated proteins per term is presented in Figure 1D (also see Supplementary Materials). Two protein groups were analyzed with the Kyoto Encyclopedia of Genes and Genomes (KEGG) Pathway Database. Proteins within the group of regulation of SMAD protein ontology were found to be relevant to the TGF-beta pathway (KEGG hsa04350). Subsequently, proteins within the group of negative regulation to oxidative stress ontology were found to be relevant to the immune network for immunoglobulin production (KEGG hsa04672). 

Beneath the protein expression analysis, it was tentatively confirmed that the secretions of M-MSCs could be more prominent in the regulation of inflammation compared to BM-MSCs. Accordingly, secretions of M-MSCs were chosen as a new AD therapeutic candidate and applied to in vivo AD models.

### 3.2. Induction and Alleviation of AD-Like Lesions on Mouse Skin

**Figure 2:**
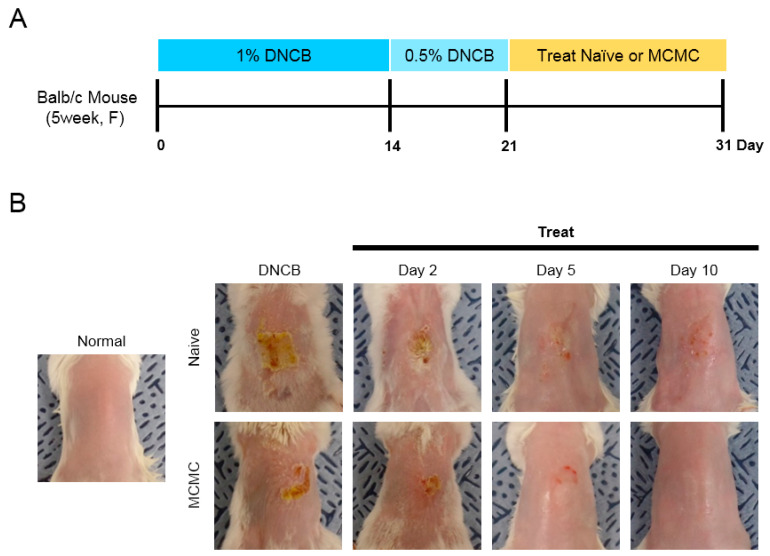
Schematic diagram of the study protocol, Photographs of the dermal skin lesions. Induction and treatment of AD (atopic dermatitis)-like skin lesions in mice. (**A**) Schematic diagram of the study protocol. To induce atopic dermatitis-like lesions, DNCB (1-chloro-2,4-dinitrobenzene) was applied ectopically to Balb/c mice. A total of 80μL of 1% DNCB in sunflower oil was applied for 2 weeks, and 80μL of 0.5% DNCB was applied for 1 week. Mice were then treated with 80μL of MCMC (M-MSC conditioned media concentrate) or naïve media for 10 days. (**B**) Representative macroscopic dorsal skin lesions. MCMC showed therapeutic effects on DNCB-induced AD-like skin symptoms.

**Figure 3:**
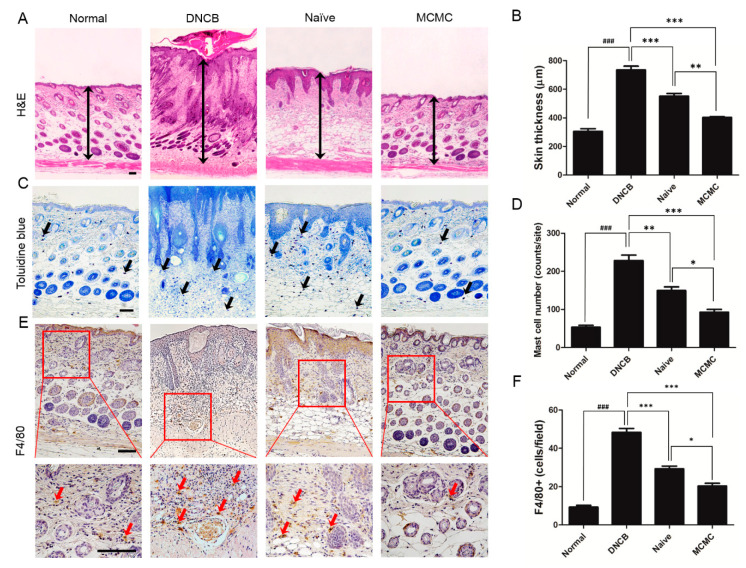
Histological analysis. Effects of MCMC on the histology of lesions in DNCB-treated mice. (**A**) Dorsal skin sections were stained with hematoxylin-eosin (H&E). Black up-down arrows indicate epidermal and dermal thickness. (**B**) The skin thickness was measured in H&E slides. (**C**) Mast cells were stained purple (black arrows) in toluidine blue-stained (TB) slides. (**D**) The number of mast cells was measured in TB slides. (**E**) Immunohistochemical (IHC) staining of F4/80 in mouse skins. Red boxes demonstrate the enlarged view and red arrows indicate dark brown dots as macrophages. (**F**) The number of macrophages was measured in IHC slides. The scale bar in Figure A is 100 µm. The other scale bars are 500 µm. Data are shown as mean±SD (*, *p* < 0.05; **, *p* < 0.01; ***, *p* < 0.001; ###, *p* < 0.001).

We established a mouse model with AD-like skin lesions as previously described (Figure 2A). These skin lesions included dryness, slime, and scab. After three weeks of induction, back skins of Balb/c mice were treated with MCMC (MCMC group) or naïve media concentrate (Naïve group) for 10 consecutive days. Compared to the DNCB group, the Naïve group had less severe lesions, but more dryness, slime, and scab were observed than the MCMC group. (Figure 2B). Their AD-like lesion alleviation was confirmed with histopathological analyses. The skin thickness was measured in H&E stained sections and compared to the DNCB group, and the other two groups showed thinner skin. In particular, the MCMC group showed significantly thinner skin than the Naïve group. (Figure 3A,B). In aspects of in vivo inflammatory cell proliferation, significantly fewer mast cells (Figure 3C,D) and macrophages (Figure 3E,F) were observed in the order of the DNCB group, Naïve group, and MCMC group. 

**Figure 4:**
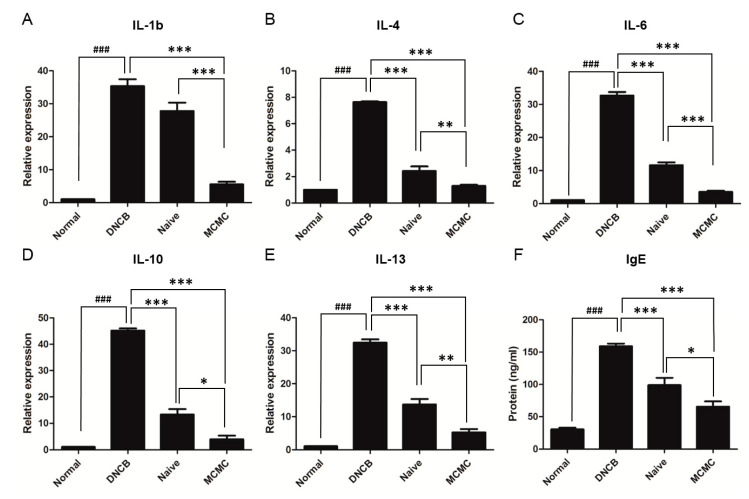
MCMC inhibits cytokine expression. Effects of MCMC on cytokine expression. (**A**–**E**) Effects of MCMC on the mRNA expression levels of inflammatory cytokines in DNCB-induced AD-like skin lesions. The DNCB group’s dorsal skin tissues of mice were cut on day 21 and the rest of the groups were cut on day 31. The relative mRNA expression levels of IL-1b, IL-4, IL-6, IL-10, and IL-13 were analyzed using quantitative PCR. (**F**) The blood samples by cardiac puncture were collected at day 21 (DNCB group), 31 (the rest of the groups). Total levels of serum IgE were measured by ELISA. Data are shown as mean ± SEM in qPCR data and as mean±SD in ELISA data (* *p* < 0.05; ** *p* < 0.01; *** *p* < 0.001; ### *p* < 0.001).

Consecutively, these tendencies also coincided within the qPCR and ELISA analyses, which were conducted for pro-inflammatory cytokines such as IL-1b, IL-4, IL-6, IL-10, and IL-13 and the serum IgE level as a systemic reaction indicator. The expression levels of the pro-inflammatory cytokines were significantly decreased in the MCMC group compared to those in the DNCB group and Naïve group (Figure 4A–E), and one of the universal markers of AD, the serum IgE levels measured with ELISA, were also significantly lower in the MCMC group than the DNCB group and Naïve group (Figure 4F).

## 4. Discussion

New breakthroughs are required in the development of new therapies for AD, which is a pruritic inflammatory skin condition caused by factors such as immune imbalance [33]. To date, several drugs have been used to relieve AD lesions, including corticosteroids and antihistamines [34]. Most of the conventional therapeutics, however, have various side effects, and some of them have not been evaluated or identified by regulatory authorities. With regard to the regulation of inflammation in AD lesions, MSCs would be worthwhile to develop as a treatment since MSCs can affect natural immune function regulation [35,36] and local inflammatory response induction [36,37]. Among MSCs, BM-MSCs have been reported to possess better anti-inflammatory potency than MSCs derived from adipose tissues or umbilical cord blood [38]. If there are other cell resources with advantages of MSCs and superiorities to BM-MSCs in aspects of therapeutic development, they are expected to contribute to the development of more effective AD therapies. 

M-MSCs might be a stronger candidate than BM-MSCs in terms of AD therapeutic development. M-MSC is derived from hESC which could be a virtually unlimited resource of therapeutic cells. M-MSC also has important advantages in terms of therapeutic development, including the ability to control simultaneous differentiation to ensure optimal safety and efficacy in the case of transplantation. The utility of M-MSCs as therapeutic agents can also be confirmed by the results of the L507 protein assay. M-MSCs had a highly moderate correlation with BM-MSCs in overall protein expression profiles, showing characteristics of MSCs. Based on these results, M-MSCs were expected to have therapeutic functional potentials as conventional MSC models do. Protein expressions related to the ontology of the regulation of SMAD protein identified in the analysis of DEPs can be understood in the same context. Proteins involved in this ontology play a role in cell-fate decisions through the regulation of the TGF-beta pathway [39]. They could contribute to the maintenance of stem cell status by regulating cell proliferation and development. 

Results of the analysis of DEPs revealed that M-MSCs had significantly higher expression levels of proteins involved in the ontology of negative regulation of the cellular response to oxidative stress than BM-MSCs. Proteins involved in this ontology could play a role in the immune network for producing immunoglobulins, neutralizing toxins, and removing pathogenic factors [40]. Since the mucosa as the first defense line of the human body plays an important role in maintaining immune homeostasis [41,42], these phenomena can be mainly observed in the mucosal immune system [43]. Based on these protein analyses, the use of M-MSCs as a therapeutic is expected to have a positive effect on the balance of immunological homeostasis.

Subsequently, to identify therapeutic potentials of the immunomodulation of M-MSCs, we used a DNCB-induced AD-like model that mimics AD inflammation in mice. As previously reported in AD patients [44], when the lesions were alleviated with the therapies the gene expression levels of inflammatory cytokines including IL-1b, IL-4, IL-6, IL10, and IL-13 and serum IgE levels were significantly decreased, which was followed by histopathological observations [4]. Among the affected cytokines, IL-4 could be a key player in the AD therapeutic field. As shown in the L507 protein analysis, M-MSCs would be significantly superior to BM-MSCs in aspects of the cell-fate regulation via the TGF-beta pathway. Given that IL-4 is known to be counterbalanced with TGF-beta and they can cancel each other out [45], it is possible that MCMC could regulate IL-4 effects on mast cell and IgE-mediated hypersensitivity reactions via TGF-beta emphasizing. M-MSCs could also regulate IL-4 expression by lowering oxidative stress [46], which is also shown as M-MSC characteristics in the L507 analysis. These results showed the possibility of developing M-MSCs as a therapeutic for AD. 

This study confirmed the properties of M-MSCs as an MSC line and presented the possibility of M-MSCs in aspects of immune regulation based on protein expression analysis. This study also revealed that M-MSC secretions have significant relief effects in an acute AD model via their anti-inflammatory effect. These results suggest that M-MSCs could be worthwhile to be developed as a next-generation treatment for AD.

## Figures and Tables

**Table 1:** Primer sequences.

Gene	Forward	Reverse
GAPDH	TCACTGCCACCCAGAAGA	GACGGACACATTGGGGGTAG
IL-1b	GCAACTGTTCCTGAACTCAACT	ATCTTTTGGGGTCCGTCAACT
IL-4	TCACTGACGGCACAGAGCTA	CTTCTCCTGTGACCTCGTT
IL-10	CAGTGGAGCAGGTGAAGAGTG	CAAGGAGTTGTTTCCGTTAGC
IL-6	TAGTCCTTCCTACCCCAATTTCC	TTGGTCCTTAGCCACTCCTTC
IL-13	TGAGGAGCTGAGCAACATCACACA	TGCGGTTACAGAGGCCATGCAATA

## Supplementary Materials

The following are available online at https://www.mdpi.com/2227-9059/8/10/439/s1, Table S1: Proteins including each node in Figure 1C,D.
