# Supplementary material for: Anti-Inflammatory Effects of M-MSCs in DNCB-Induced Atopic Dermatitis Mice
Source: Biomedicines. 2020 Oct 21;8(10):439. doi: 10.3390/biomedicines8100439 (PMC7589030; doi:10.3390/biomedicines8100439)
Supplement: Supplementary file 1 [file biomedicines-08-00439-s001.pdf]

Supplementary Materials

Table S1. Proteins including each node in Fig. 1(C) and Fig. 1(D).

#	GO Term	% Associated Genes	# of Genes	Associated Genes Found
**1**	alpha-beta T cell activation involved in immune response	4.347826	3	[IFNG, IL2, IL6]
**2**	alpha-beta T cell differentiation involved in immune response	4.347826	3	[IFNG, IL2, IL6]
**3**	CD4-positive, alpha-beta T cell differentiation involved in immune response	4.411765	3	[IFNG, IL2, IL6]
**4**	negative regulation of cytokine biosynthetic process	8.333333	3	[IL10, IL6, INHBB]
**5**	negative regulation of response to drug	6	3	[IL10, IL6, MET]
**6**	cell death in response to hydrogen peroxide	5.555555	3	[IL10, IL6, MET]
**7**	regulation of hydrogen peroxide-induced cell death	6	3	[IL10, IL6, MET]
**8**	regulation of response to reactive oxygen species	4.918033	3	[IL10, IL6, MET]
**9**	negative regulation of hydrogen peroxide-induced cell death	8.333333	3	[IL10, IL6, MET]
**10**	negative regulation of cellular response to drug	8.333333	3	[IL10, IL6, MET]
**11**	regulation of cellular response to drug	5.454545	3	[IL10, IL6, MET]
**12**	negative regulation of cellular response to oxidative stress	4	3	[IL10, IL6, MET]
**13**	negative regulation of response to reactive oxygen species	8.333333	3	[IL10, IL6, MET]
**14**	negative regulation of oxidative stress-induced cell death	4	3	[IL10, IL6, MET]
**15**	cytokine activity	4.642857	13	[BMP2, CXCL1, CXCL8, EML2, GDF1, GDF3, IFNG, IL10, IL2, IL6, INHBB, MSTN, TNFSF8]
**16**	growth factor activity	4.878049	10	[BMP2, CXCL1, FGF19, GDF1, GDF3, IL10, IL2, IL6, INHBB, MSTN]
**17**	regulation of pathway-restricted SMAD protein phosphorylation	9.459459	7	[ACVR1, ACVR2A, BMP2, GDF1, GDF3, INHBB, MSTN]
**18**	positive regulation of osteoblast differentiation	6.756757	5	[ACVR1, ACVR2A, ACVR2B, BMP2, IL6]
**19**	positive regulation of pathway-restricted SMAD protein phosphorylation	12.5	7	[ACVR1, ACVR2A, BMP2, GDF1, GDF3, INHBB, MSTN]
**20**	pathway-restricted SMAD protein phosphorylation	9.090909	7	[ACVR1, ACVR2A, BMP2, GDF1, GDF3, INHBB, MSTN]
**21**	SMAD protein signal transduction	4.854369	5	[BMP2, GDF1, GDF3, INHBB, MSTN]
**22**	positive regulation of epithelial to mesenchymal transition	5.357143	3	[ACVR1, BMP2, IL6]
**23**	activin receptor signaling pathway	6.060606	4	[ACVR1, ACVR2A, ACVR2B, INHBB]

**24**	positive regulation of bone mineralization	8	4	[ACVR1, ACVR2A, ACVR2B, BMP2]
**25**	positive regulation of transmembrane receptor protein serine/threonine kinase signaling pathway	5.797101	8	[ACVR1, ACVR2A, ACVR2B, BMP2, GDF1, GDF3, INHBB, MSTN]
**26**	positive regulation of ossification	4.716981	5	[ACVR1, ACVR2A, ACVR2B, BMP2, IL6]
**27**	activin-activated receptor activity	33.33333	3	[ACVR1, ACVR2A, ACVR2B]
**28**	regulation of activin receptor signaling pathway	6.976744	3	[ACVR1, ACVR2A, ACVR2B]
**29**	regulation of bone mineralization	4.210526	4	[ACVR1, ACVR2A, ACVR2B, BMP2]
**30**	positive regulation of biomineral tissue development	6.666667	4	[ACVR1, ACVR2A, ACVR2B, BMP2]
**31**	gastrulation with mouth forming second	12.12121	4	[ACVR1, ACVR2A, ACVR2B, GDF3]
**32**	transforming growth factor beta-activated receptor activity	18.75	3	[ACVR1, ACVR2A, ACVR2B]
**33**	transmembrane receptor protein serine/threonine kinase activity	15.78947	3	[ACVR1, ACVR2A, ACVR2B]
